# Advantages of Cubosomal Formulation for Gatifloxacin Delivery in the Treatment of Bacterial Keratitis: In Vitro and In Vivo Approach Using Clinical Isolate of Methicillin-Resistant *Staphylococcus aureus*

**DOI:** 10.3390/ma15093374

**Published:** 2022-05-08

**Authors:** Mohamed Nasr, Sameh Saber, Alaa Y. Bazeed, Heba A. Ramadan, Asmaa Ebada, Adela Laura Ciorba, Simona Cavalu, Heba I. Elagamy

**Affiliations:** 1 Department of Pharmaceutics, Faculty of Pharmacy, Delta University for Science and Technology, Gamasa 35712, Egypt; alaayosf@gmail.com (A.Y.B.); hebaelagamy1985@yahoo.com (H.I.E.); 2 Department of Pharmaceutics and Industrial Pharmacy, Faculty of Pharmacy, Helwan University, Cairo 11790, Egypt; 3 Department of Pharmacology, Faculty of Pharmacy, Delta University for Science and Technology, Gamasa 35712, Egypt; sampharm81@gmail.com; 4 Department of Microbiology and Immunology, Faculty of Pharmacy, Delta University for Science and Technology, Gamasa 11152, Egypt; Hebaaa.aadel@gmail.com (H.A.R.); asmaaebada89@yahoo.com (A.E.); 5 Faculty of Medicine and Pharmacy, University of Oradea, 410087 Oradea, Romania; simona.cavalu@gmail.com

**Keywords:** gatifloxacin, cubosomal dispersion, MIC, corneal permeation, methicillin resistant *Staphylococcus aureus*, keratitis

## Abstract

The objective of this study was to enhance the corneal permeation of gatifloxacin (GTX) using cubosomal nanoparticle as a delivery system. Cubosomal nanoparticle loaded with GTX was prepared and subjected for in vitro and in vivo investigations. The prepared GTX-loaded cubosomal particles exhibited nanoparticle size of 197.46 ± 9.40 nm and entrapment efficiency of 52.8% ± 2.93. The results of ex vivo corneal permeation of GTX-loaded cubosomal dispersion show approximately 1.3-fold increase compared to GTX aqueous dispersion. The incorporation of GTX into cubosomal particles resulted in a fourfold reduction in the minimum inhibitory concentration (MIC) value for the GTX cubosomal particles relative to GTX aqueous dispersion. Furthermore, the enhanced corneal penetration of GTX-loaded cubosomal dispersion compared was evident by a significant decrease in the area % of corneal opacity in MRSA infected rats. Moreover, these results were confirmed by photomicrographs of histological structures of corneal tissues from rats treated with GTX-cubosomal dispersion which did not present any change compared to that of the normal rat corneas. In conclusion, treatment of ocular bacterial infections and reduction in the probability of development of new resistant strains of MRSA could be accomplished with GTX-loaded cubosomal nanoparticles.

## 1. Introduction

The ocular drug delivery field is considered one of the most attractive, inspiring, and competitive fields owing to the many obstacles found in drug application due to the complex physiological and chemical nature of the eye [1,2]. Recently, many formulation strategies have been implemented to evolve new drug delivery systems which can overcome the inadequacies of the conventional ocular delivery systems including poor formula retention time, pulsed dosing of drug, frequent drug administration which leads to mechanical injury, corneal pigmentation, as well as sensitivity reactions of the conjunctiva and large drainage factor which leads to low ocular bioavailability [3,4,5].

The previously mentioned factors demand the development of a novel ocular drug delivery system that would extend the pre-ocular retention, and hence enhance the ocular penetration and absorption of drugs through ocular barriers providing new tools for the therapy of anterior and posterior eye segment diseases [6]. Novel ocular delivery systems based on nanoparticles have recently become alternative tools for traditional ocular delivery systems such as polymeric nanoparticles [7,8,9], polymeric micelles [10], solid lipid nanoparticles [11], nanostructured lipid carrier [12] and nano-emulsion [13].

Gatifloxacin (GTX) is a fourth generation fluoroquinolone antibacterial used for treatment of ocular infections through inhibiting bacterial DNA replication, transcription and repair [14,15]. Compared to other quinolones, GTX is more effective with broader spectrum especially in the case of staphylococcus and streptococcus infections [16]. However, the use of GTX in ocular infections is limited by its sub-therapeutic concentrations in the corneal aqueous humour and iris-clarity body due to the reduced GTX permeability. The lower GTX permeability might be attributed to efflux pumps such as P-glycoprotein (P-gp/MR1/ABCB1) and multi-drug resistance-associated protein 2 (MRP2/ABCC2/cMOAT) [17]. The multi-drug resistance associated with these efflux pumps may limit the drug concentration at the target site and, subsequently, reduce drug uptake by the bacteria. Moreover, GTX suffers from pH-dependent solubility with maximum solubility ranging from 40 to 60 mg/mL at pH 2–5 [18]. These limitations necessitate the development of a novel drug delivery system rather than the traditional ophthalmic eye drops solution.

Glyceryl-monooleate (GMO) is used to form novel self-assembled thermodynamically stable cubic nanoparticles known as cubosomes which are stabilized using poloxamer 407 (P407). Cubosomes, as dispersed colloidal particles of cubic phase liquid crystals, have drawn attention to their use in controlled-release drug delivery systems. Cubosomes are advantageous due to their simple formulation and high loading capacity for different drugs with different nature. Moreover, extended drug release, biocompatibility with biological tissues, bio-adhesion, exceptional penetration ability and biodegradability give this drug delivery system superiority in ocular delivery. These features were previously reported upon employing cubosomes for many drugs such as dexamethasone and fluconazole as an ophthalmic drug delivery system [19,20].

Hence, the objective of the current study is to investigate the potential of cubosomal nanoparticles dispersion as a promising drug carrier for ocular administration of GTX for enhancing in vitro and in vivo performance of the drug. GTX-loaded cubosomal dispersions were in vitro evaluated for particle size, shape, drug entrapment, and ex vivo trans-corneal permeation characteristics using isolated rabbit cornea. The extent of treatment of induced bacterial keratitis in rats was used to evaluate the in vivo performance of the drug-loaded cubosomes compared to free drug dispersion. The cubosomal formulation is expected to improve the permeability of GTX and decrease the incidence of its resistance.

## 2. Materials and Methods

### 2.1. Materials

Pure GTX was gifted by Delta Grand Pharma for pharmaceutical industries (10th of Ramadan City, Egypt). Glyceryl monooleate (GMO) was supplied by Gattefosse, Lyon, France under the trade name Paceol^®^. Poloxamer 407 (P407) was acquired from Sigma-Aldrich (Milwaukee, WI, USA). Acetonitrile (HPLC grade) was purchased from VWR International Ltd., Poole, England. Potassium dihydrogen phosphate, sodium hydroxide, disodium hydrogen phosphate (pharmaceutical grade), phosphoric acid, and methanol (analytical grade) was bought from El Nasr, Cairo, Egypt. 

### 2.2. GTX-Loaded Cubosomes Formulation

An emulsification technique was used to prepare the drug-loaded cubosomal dispersion reference to Nasr et al. [21]. At the beginning, GMO was melted at 60 °C by heating in a water bath. Then, 2 mL of deionized water was added drop wise with continuous stirring until homogenous gel formation. The obtained gel was left for 24 h at room temperature. After which, a solution of GTX and 10% *w*/*w* of P407 (relative to GMO) in phosphate buffer (pH 5) was added to the gel using a high-speed vortex for 3 min. Probe sonication (Sonics vibra cell, Sonics & Materials INC, Newtown, CT, USA) was used in the obtained dispersion for 5 min (5 s on and 3 s off). The final weight of the dispersion was adjusted to 50 g using deionized water. The final GTX concentration in the obtained dispersion was 3 mg/g.

### 2.3. Particle Size Analysis, Polydispersity Index and Zeta Potential

A Zetasizer Nano series (Nano ZS, Malvern, UK) was implemented to measure the particle size distribution, polydispersity index (PDI) and zeta potential of cubosomal nanoparticles at 25 ± 0.5 °C in triplicate after being properly diluted with deionized water.

### 2.4. Transmission Electron Microscopy (TEM)

The morphology of GTX-cubosomal nanoparticles was observed by a transmission electron microscope (JEOL, Tokyo, Japan), model JEM-2100 with super twin lens. A drop of the cubosomal dispersion was put on carbon-coated copper grid and 1% sodium phosphotungstate solution as a stain. The stained sample was left to dry for 15 min at room temperature before monitoring.

### 2.5. Entrapment Efficiency (EE %)

Amicon Ultracentrifuge tubes (3000 MWCO, Millipore, St. Louis, MO, USA) were utilized to measure the entrapment efficiency by ultrafiltration centrifugation technique [22] as follows: 9 mL of deionized water was used to dilute 1 mL of GTX-loaded cubosomes, subsequently, a sample of 3 mL of the diluted dispersion was centrifuged at 6000 rpm for 10 min. Deionized water was used to properly dilute the acquired filtrate and measured spectrophotometrically at 290 nm (Jenway, Stafford, UK) [23] representing free GTX (Q Free). To determine the total amount of GTX existing in 1 mL cubosomal dispersion (Q Total), the formulation was appropriately diluted with methanol and measured spectrophotometrically as mentioned above using methanol as blank. EE % was calculated according to the following equation:

EE % = [(Q Total − Q Free) / Q Total] × 100(1)

### 2.6. Differential Scanning Calorimetry (DSC)

A thermal analysis study was conducted on the cubosomal dispersion, each of its components separately and with pure GTX. The DSC thermograms were achieved using DSC-60, Shimadzu, Kyoto, Japan. The aluminum pan was filled with 5 mg of each sample, heated with a 10 °C/min heating rate under a purgative nitrogen. A comparable empty pan was used as the control.

### 2.7. Ex Vivo Corneal Permeation Study

The Delta university ethics committee authorized the protocol of study (Approval Number: FPDU11621/3). Rabbits’ corneas were utilized in this experiment after being freshly separated and submerged for 30 min in fabricated lacrimal fluid before the experiment. The corneas were mounted onto one end of a plastic syringe imitating a dialyzing tube with permeation area of nearly 1.54 cm^2^ using a rubber band to ensure that they were watertight. The surface epithelium layer of each cornea was placed inward of the syringe, while the deepest endothelium layer was directed outwards facing the permeation medium. An equivalent amount (3 mg) of pure and tested formula was placed on the donor compartment. The acceptor compartment (50 mL of phosphate buffer of a pH 7.4 and a temperature of 37 ± 0.5 °C) was stirred at 50 rpm. The corneal surface of the donor compartment was adjusted just to touch the surface of permeation medium. At different time intervals (30 min, 1, 2, 3, 4, 5, 6, 8, 10, 11 and 12 h), 1 mL of the permeation media was removed, and an equivalent volume was used to replenish it. Withdrawn samples were analyzed using HPLC after being passed through a 0.45 μm syringe filter. The cumulative amount of GTX permeated per unit area was calculated and plotted versus time. The slope of the plot was calculated and taken as flux (mg/cm^2^/h).

### 2.8. HPLC Assay of GTX

The HPLC technique was implemented using Shimadzu CTO-20A (Kyoto, Japan) with UV detector (VWD 1260) and ODS Hypersil column, with an average particle size of 5 μm, 150 mm tall and 4.6 mm internal diameter (Thermo Fisher Scientific, Waltham, MA, USA). Corresponding to a previously reported method [24] with minor changes modifications, acetonitrile and potassium phosphate buffer (pH 5) were used as a mobile phase with ratio (65:35 *v*/*v*), respectively. An isocratic elution was used with flow rate 1.5 mL/min and injection volume of 20 μL. The UV detector was set at 290 nm and 254 nm. The method was validated with reasonable linearity in the range of 1–8 µg/mL with 0.995 correlation coefficient. In addition, all samples were assayed for interday and intraday accuracy and precision.

### 2.9. Determination of Minimum Inhibitory Concentration (MIC) of GTX

The in vitro antibacterial activity of GTX-loaded cubosomal formulation against clinical isolate of methicillin-resistant Staphylococcus aureus (MRSA) was investigated compared to GTX aqueous dispersion using MIC test. Broth microdilution technique was employed for determination of MIC as described by Andrews [25] and according to CLSI M07-A9 guidelines [26]. The test was performed in 96-well rounded bottom microtiter plate filled with 100 μL of Muller Hinton broth medium. Two-fold serial dilutions of both treatments were prepared using sterile distilled water. The tested concentrations of GTX were ranged from 0.03 to 16 μg/mL. Aliquots (100 μL) of each concentration were loaded in 96-well flat-bottom microtiter plates. The bacterial inoculums were adjusted to the concentration of 5 × 105 CFU/mL. Positive and negative GTX free controls were formulated in the presence and absence of bacteria, respectively. All plates were incubated at 37 °C for 18 h. The lowest concentration that inhibits the bacterial growth is considered as the minimum inhibitory concentration.

### 2.10. In Vivo Studies

The in vivo study was conducted to assess the influence of cubosomal formulation on the antibacterial activity of GTX against methicillin-resistant strain (MRSA). Staphylococcus aureus is the main infective organism causing endophthalmitis and in some cases vision loss due to keratitis [27]. Clinical isolate of MRSA, isolated from a human corneal ulcer (Outpatient clinics, Mansoura University Ophthalmic Hospital, Egypt), was utilized as a pathogen to establish infection. Briefly, one colony of bacterial isolate was picked up from a fresh culture plate and injected into 5 mL of Luria Broth media, which was then incubated at 37 °C for 24 h with constant shaking at 150 rpm. The growth suspension was diluted until turbidity was matched with 0.5 McFarland standards to obtain approximately the organism number of 1 × 108 colony forming units (CFU) per mL.

Twenty-four adult male Wistar rats (270 ± 10 g) were enrolled in the study. The study and its procedures were accepted by the Institutional Animal Care and Use Committee (IACUC) at the Faculty of Pharmacy Delta University (Approval Number: FPDU11621/3) and were performed in agreement with relevant guidelines and regulations.

For all rats to be immunocompromised, they were treated with intraperitoneal injection of methyl prednisolone at a dose of 50 mg/kg in saline (300 µL/rat) for 3 consecutive days to facilitate the development of bacterial infection. Four groups (6 rats each) were divided as follows: group 1 (uninfected untreated rats); group 2 (infected rats with no treatment); group 3 (infected rats treated with GTX aqueous solution); and group 4 (infected rats treated with GTX-loaded cubosomal dispersion). A 27-gauge needle was used to scare the cornea of the right eye of each rat. Tear film break up was accomplished by pipetting 10 µL of 0.6% acetylcysteine onto the cornea and after that, normal saline solution was used for washing. After which, a 10 µL MRSA suspension (5 × 108 CFU) was applied and spread evenly. The left eyes of rats were scarred by the same pattern but were not subjected to infection to serve as a control.

The treatment began three days after infection. An ophthalmologist, who was blinded to the treatment protocol, confirmed the infection. A clear visual cloudy cornea was noticed at the 3rd day after the infection. Treatments were resumed for another 2 days until the vanishing of the cornea’s cloudy appearance in one rat from treatment groups. On the 5th day after infection, the photographs of the affected eyes were processed using ImageJ 1.52i software (NIH, Bethesda, MD, USA). Images were captured at a 35 cm distance and processed using the lower threshold level at 95 and the upper threshold level at 145.

### 2.11. Histological Examination

At the end of the in vivo study, cervical dislocation was applied for euthanization of animals. From each group, some rats’ eyes served as an autopsy sample where it was fixed in 10% formalin solution for 24 h, then rinsed with distilled water. Afterwards, dehydration was accomplished by using different concentrations of alcohol. Xylene was used for washing specimens followed by embedding it in paraffin at 56° in an oven for 24 h. Tissue paraffin beeswax was cut into blocks 4 μm in thickness using Sledge microtome. The final tissue sections were and stained by hematoxylin and eosin stain after removal of paraffin and examined by the light electric microscope [28].

### 2.12. Statistical Analysis

Differences between groups were analyzed by one-way analysis of variance followed by Tukey as a Post-Hoc Test using GraphPad Prism software, 8.0.2 (GraphPad Software Inc., La Jolla, CA, USA). Data are presented as the mean ± standard deviation (SD) (*p* ≤ 0.05).

## 3. Results and Discussion

### 3.1. Particle Size, Polydispersity Index and Zeta Potential

The formed cubosomal dispersion displayed a narrow monomodal with mean particle size of 197.46 ± 9.40 nm. The PDI value of the cubosomal dispersion was 0.14 ± 0.05 indicating acceptable homogeneity. The zeta potential of the cubosomal nanoparticles was −21.90 ± 2.03 mV indicating high stability and lower tendency for particles aggregation.

### 3.2. Morphology of GTX-Cubosomal Nanoparticles

**Figure 1:**
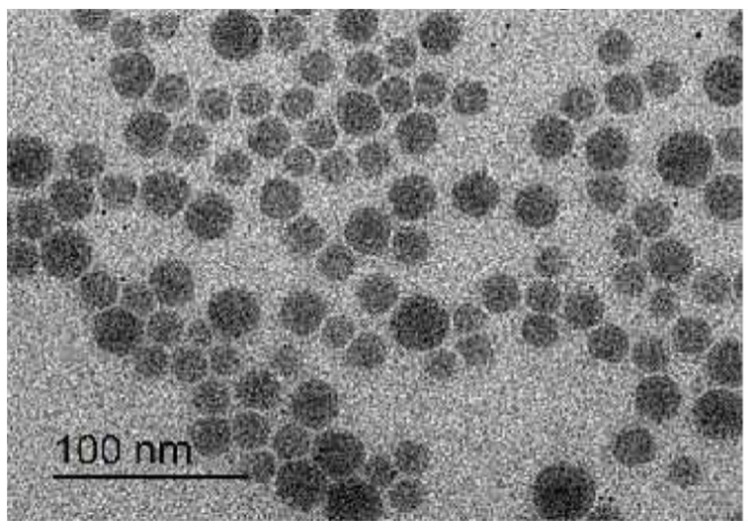
TEM photomicrograph of GTX loaded cubosomes.

The TEM photomicrograph of GTX-cubosomal nanoparticles (Figure 1), revealed that the GTX-loaded cubosomal particles are nearly spherical polyangular non-aggregated particles with homogenous and a narrow size distribution. However, diameters of TEM photographed particles are smaller relative to the measured particle size by the dynamic light-scattering particle size analyzer.

### 3.3. Entrapment Efficiency

The EE % of GTX in cubosomal nanoparticle was 52.8% ± 2.93. The relatively lower % of drug entrapment might be attributed to the hydrophilic nature of GTX (log *p* = −0.23) and the higher % of the ionized form of GTX in phosphate buffer pH 5.5 that may lead to lower partitioning of the drug into the cubosomal lipid bilayer and favor the leakage of the drug from the water channels of cubosomal particles.

### 3.4. Differential Scanning Calorimetry

**Figure 2:**
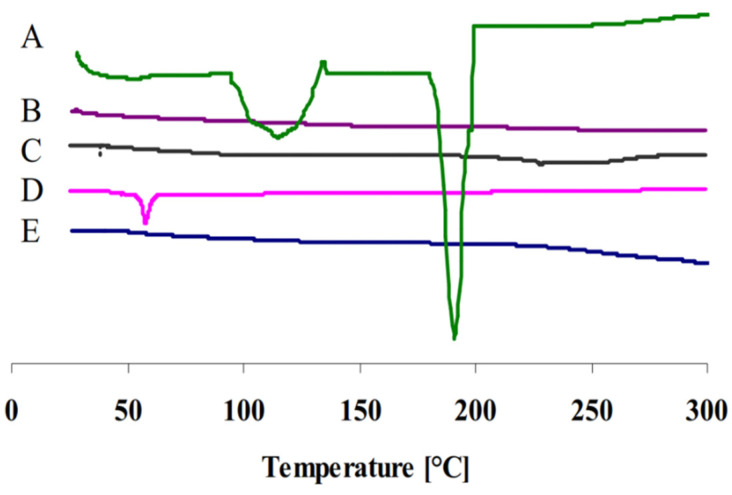
DSC thermograms of (A) pure GTX, (B) GTX-loaded cubosomal dispersion, (C) blank cubosomal dispersion, (D) P407 and (E) GMO.

Figure 2 represents the thermograms of pure GTX, GTX-loaded cubosomal dispersion, blank cubosomal dispersion, P407 and GMO. The pure GTX powder presented a thermogram with a broad endothermic peak at 100–150 °C which may be due to dehydration and some structure rearrangement of GTX and a sharp endothermic peak of 191 °C indicating its crystalline nature [29]. In addition, the sharp characteristic peak disappeared in the thermogram of cubosomal dispersion indicating that GTX was not in a crystalline state but rather present in an amorphous state.

### 3.5. Ex Vivo Corneal Permeation

**Figure 3:**
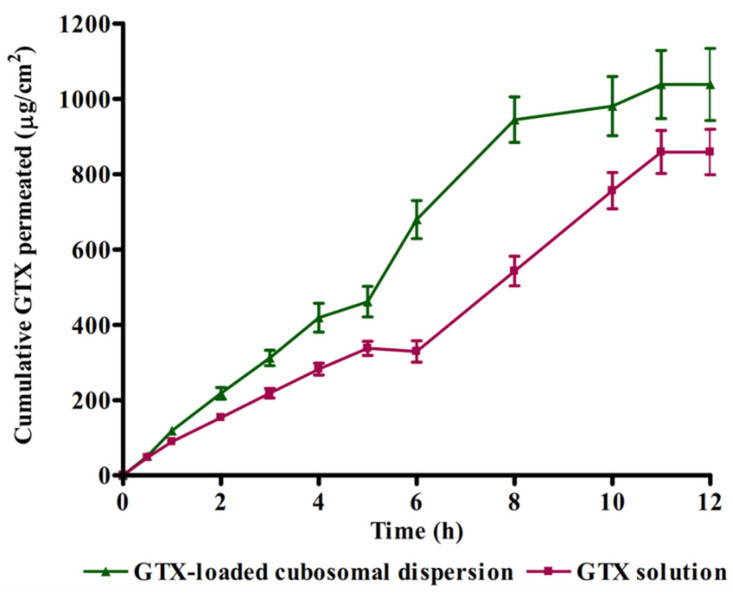
Comparison between rabbit corneal permeation of GTX delivered from cubosomal dispersion and aqueous GTX dispersion.

Figure 3 illustrates the result of the rabbit corneal permeation of GTX from cubosomal dispersion compared to aqueous GTX dispersion. The cubosomal dispersion showed approximately a 1.3-fold increase in GTX corneal permeation. The slope of the graph (flux) of the cubosomal dispersion was significantly (*p* < 0.05) higher in the case of cubosomal dispersion (93.84 ± 7.89 μgcm^−2^ h^−1^) in comparison with GTX aqueous dispersion (73.01 ± 3.45 μgcm^−2^ h^−1^). The enhanced permeability could be attributed to the bio-adhesive ability of cubosomes to attach to the large epithelium surface with lipophilic nature which will improve the delivery of the drug [30]. In addition, the cubosomal structure has similar features as the natural biological membrane which might facilitate drug permeability and consequently drug absorption. Besides, the components of cubosomal nanoparticles (GMO and P407) may play an important role in alteration of the activity of P-glycoprotein efflux pumps by both membrane permeability changes through a reduction in membrane micro-viscosity and depletion of ATP levels [31]. These results agree with previously reported studies that utilized cubosomes as delivery systems for enhanced ocular delivery of fluconazole, flurbiprofen and sertaconazole [20,32,33].

### 3.6. Influence of GTX-Loaded Cubosomal Nanoparticles on MIC

MICs of GTX-loaded cubosomal dispersion and free GTX aqueous dispersion were determined to evaluate the influence of cubosomal formulation on values of MIC against MRSA. The values of MIC were 4 and 16 µg/mL for GTX-loaded cubosomal dispersion and GTX aqueous dispersion, respectively. The result reveals that the incorporation of GTX into cubosomal particles resulted in a significant reduction in MIC value. The fourfold reduction in MIC value of GTX cubosomal formulation is expected to enhance the antibacterial activity of GTX and reduce the chance for development of new resistant bacterial strains. The obtained results could be attributed to the composition of cubosomal particles with the presence of GMO as a penetration enhancer in addition to its bioadhesiveness that facilitate a possible adhesion and/or fusion to bacterial cell membrane and consequently improve the drug permeability into bacterial cells [34].

### 3.7. In Vivo Studies

#### 3.7.1. Efficacy of GTX-Loaded Cubosomal Nanoparticles in the Treatment of MRSA-Induced Ocular Keratitis

**Figure 4:**
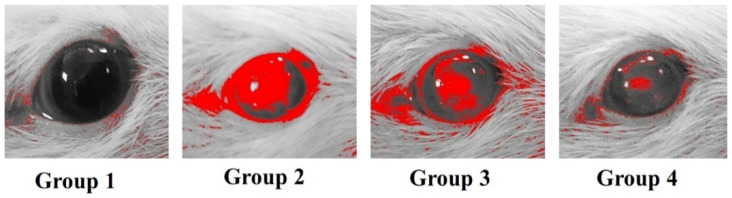
ImageJ-processed photographs of eyes from different groups: group 1 (uninfected untreated rats); group 2 (infected rats without treatment); group 3 (rats treated with GTX aqueous dispersion); and group 4 (rats treated with GTX-loaded cubosomal dispersion). The red color in the processed pictures designates the areas of focal lesions.

**Figure 5:**
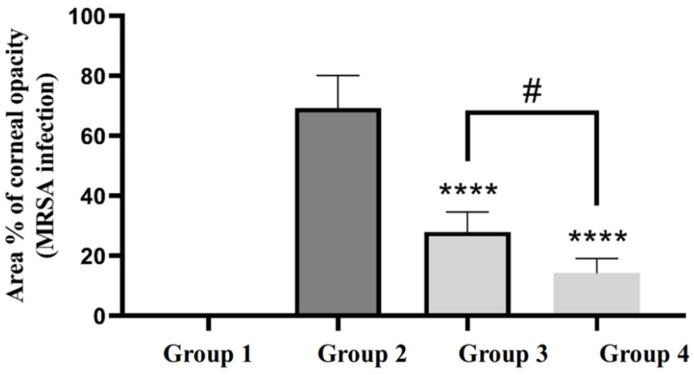
The area % of corneal opacity in different groups of rats. ****, group 4 and 3 compared to group 2 (*p* < 0.0001); #, group 4 compared to group 3 (*p* < 0.05).

Figure 4 shows the ImageJ-processed photographs of eyes from different groups. The areas of focal lesions were indicated in the processed pictures by red color. In addition, the area % of corneal opacity was calculated and considered a measure of the intensity of corneal MRSA infection (Figure 5). Statistical analysis of the obtained results revealed that GTX-loaded cubosomal dispersion significantly reduced the area % of corneal opacity compared to GTX solution. This result may be explained based on the enhanced corneal tissue penetration of GTX-loaded cubosomal nanoparticles, as previously indicated in the in vivo permeation study.

#### 3.7.2. Histological Evaluation

**Figure 6:**
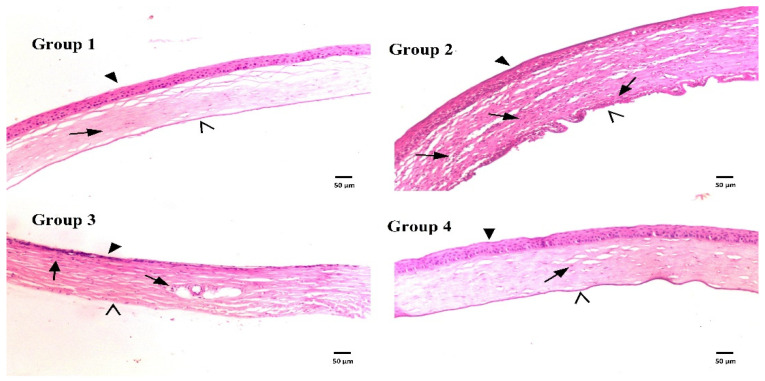
Histological structures of rat corneas stained with H&E of normal rat corneas (group 1), untreated rat corneas infected with MRSA (group 2), rat corneas infected with MRSA and treated with GTX aqueous dispersion (group 3) and rat corneas infected with MRSA and treated with GTX-loaded cubosomal dispersion (group 4).

Figure 6 displays photomicrographs of corneal tissue sections stained with H&E from different groups. The photograph of group 1 (uninfected untreated) showed normal histological structure of rat corneal epithelium (filled arrowhead), stromal connective tissue layer with no inflammatory-cell infiltration (arrow), and endothelial layer (open arrowhead). However, corneal tissue sections from infected untreated rats (group 2) showed a dense focal stratified epithelial layer (filled arrowhead), a stromal layer with apparent edema, fibrotic changes, massive inflammatory-cell infiltration (arrows), and a dense focal endothelial layer (open arrowhead). The corneal tissue sections from rats of group 3 and 4 treated with GTX dispersion or GTX-loaded dispersion, respectively, showed a lower extent of focal stratification of the epithelial layer (filled arrowhead), few or absent lymphocyte infiltration (arrows), lower degree or absence of edema in the stromal layer and normal endothelium (open arrowhead). The histological presentation of corneal tissues from rats treated with GTX-cubosomal dispersion was almost more or less similar to that of the normal rat corneas. These findings were previously illustrated upon calculation of area % of corneal opacity.

## 4. Conclusions

Incorporating GTX in cubosomal nanoparticles comprised of GMO and P407 as a stabilizer improved both in vitro and in vivo performance compared to GTX aqueous dispersion. The size of prepared formula was in the nano-range with a relatively high GTX entrapment. In vivo and in vitro experiments demonstrated that cubosomal formulation achieved higher corneal permeation and a fourfold reduction in the MIC of GTX against clinically isolated MRSA strain. The results reveal that cubosomal nanoparticle may be a promising delivery system for GTX for the treatment of ocular bacterial infections and reduce the probability of development of new resistant strains of MRSA.

## Data Availability

Data will be available upon request.
